# Understanding the neural mechanisms of lisdexamfetamine dimesylate (LDX) pharmacotherapy in Binge Eating Disorder (BED): a study protocol

**DOI:** 10.1186/s40337-019-0253-3

**Published:** 2019-07-11

**Authors:** Kristi R. Griffiths, Jenny Yang, Stephen W. Touyz, Phillipa J. Hay, Simon D. Clarke, Mayuresh S. Korgaonkar, Linette Gomes, Gail Anderson, Sheryl Foster, Michael R. Kohn

**Affiliations:** 1 The Brain Dynamics Centre, Westmead Institute for Medical Research, The University of Sydney, 176 Hawkesbury Road, Westmead, NSW 2145 Australia; 2 School of Psychology, The University of Sydney, Camperdown, NSW 2050 Australia; 3 Translational Health Research Institute, School of Medicine, Western Sydney University, Penrith, NSW 2751 Australia; 4 Adolescent & Young Adult Medicine, Westmead Hospital, Westmead, NSW 2145 Australia; 5 Centre for Research into Adolescents Health, Westmead, NSW 2145 Australia; 6 Department of Radiology, Westmead Hospital, Westmead, NSW Australia; 7 The Discipline of Medical Radiation Sciences, Faculty of Health Science, The University of Sydney, Camperdown, NSW Australia

**Keywords:** Binge eating disorder, Clinical trials, Drug therapy, Neuroimaging

## Abstract

**Background:**

The efficacy and safety of Lisdexamfetamine dimesylate (LDX) in the treatment of moderate to severe binge eating disorder (BED) has been demonstrated in multiple randomised clinical trials. Despite this, little is known about how LDX acts to improve binge eating symptoms. This study aims to provide a comprehensive understanding of the neural mechanisms by which LDX improves symptoms of BED. We hypothesise that LDX will act by normalising connectivity within neural circuits responsible for reward and impulse control, and that this normalisation will correlate with reduced binge eating episodes.

**Methods:**

This is an open-label Phase 4 clinical trial of LDX in adults with moderate to severe BED. Enrolment will include 40 adults with moderate to severe BED aged 18–40 years and Body Mass Index (BMI) of 20–45 kg/m^2^, and 22 healthy controls matched for age, gender and BMI. Clinical interview and validated scales are used to confirm diagnosis and screen for exclusion criteria, which include comorbid anorexia nervosa or bulimia nervosa, use of psychostimulants within the past 6 months, and current use of antipsychotics or noradrenaline reuptake inhibitors. Baseline assessments include clinical symptoms, multimodal neuroimaging, cognitive assessment of reward sensitivity and behavioural inhibition, and an (optional) genetic sample. A subset of these assessments are repeated after eight weeks of treatment with LDX titrated to either 50 or 70 mg. The primary outcome measures are resting-state intrinsic connectivity and the number of binge eating episodes. Analyses will be applied to resting-state fMRI data to characterise pharmacological effects across the functional connectome, and assess correlations with symptom measure changes. Comparison of neural measures between controls and those with BED post-treatment will also be performed to determine whether LDX normalises brain function.

**Discussion:**

First enrolment was in May 2018, and is ongoing. This study is the first comprehensive investigation of the neurobiological changes that occur with LDX treatment in adults with moderate to severe BED.

**Trial registration:**

ACTRN12618000623291, Australian and New Zealand Clinical Trials Registry URL: https://www.anzctr.org.au/Trial/Registration/TrialReview.aspx?id=374913&isReview=true. Date of Registration: 20 April 2018.

## Background

Binge Eating Disorder (BED) is an eating disorder characterised by recurrent episodes of excessive eating with a sense of lack of control over eating. These recurrent episodes need to occur at least once a week for 3 months to meet the diagnostic criteria of the *Diagnostic and Statistical Manual of Mental Disorders* (5th ed) [1]. It is the most common type of eating disorder in Australia, with an estimated 3-month point prevalence of 5.6%, compared with 0.5% for anorexia nervosa and 0.7% for bulimia nervosa [2]. It is also distinct from obesity, whereby obese people with BED are reported to have greater body image disturbance, lower self-esteem, and more psychiatric comorbidity with mood and anxiety disorders, compared to those without [3, 4].

Current evidence suggests that pathologic overeating in BED may be related to dysfunction of the dopamine (DA) and noradrenaline (NA) systems, increased reward sensitivity, and impulsivity towards food intake [5]. For instance, in a positron emission tomography (PET) study, high Body Mass Index (BMI) individuals with BED were found to have significantly increased DA levels in the caudate and putamen in response to food stimuli, relative to high BMI individuals without BED [6]. This significantly correlated with binge eating severity but not with BMI. Functional magnetic resonance imaging (fMRI) studies have broadly shown dysfunction in the fronto-striatal regions relating to reward and inhibition [7–12]. Further, those with BED may have impaired flexibility in reward-based decision making, with reduced activation in regions implicated in goal-directed action and evaluation of reward-based choice such the bilateral anterior insula and ventro-lateral prefrontal cortex [13]. Despite these important insights into the neuropathophysiology of BED, more studies are required to better understand neural dysfunction in BED, and how pharmacotherapies influence these systems to reduce BED symptoms.

In early 2015, the United States Food and Drug Administration (FDA) approved lisdexamfetamine dimesylate (LDX) (Vyvanse) for the treatment of moderate to severe BED in adults. Although psychotherapy is still the first-line treatment, pharmacological treatments such as LDX can be useful for those who are non-responsive to psychotherapy or for those in locations where psychotherapy is unavailable. LDX is a central nervous system stimulant, which is hydrolysed in the blood to yield long-acting d-amphetamine [14]. It increases DA and NA levels by inhibiting reuptake into the presynaptic neuron and therefore increasing the release of these monoamines into the extra neuronal space. The efficacy and safety of LDX in the treatment of moderate to severe BED for up to 12 months has been demonstrated in multi-site, double-blind, randomised, placebo-controlled clinical trials [15–19]. While reported to be generally safe, common adverse events include dry mouth, headache and insomnia [15, 16, 18], in line with other long-acting psychostimulant medications.

Despite its demonstrated efficacy for moderate to severe BED, the exact mechanism of action by which LDX improves binge eating symptoms remains unknown. Behaviorally, LDX has been shown to improve impulse control in people with BED and Attention Deficit Hyperactivity Disorder (for which LDX is also indicated) [20–22]. It is presumed that, similar to other psychostimulants, LDX improves impulse control by modulation of the prefrontal cortex, a region involved in self-regulation and inhibitory control [23, 24]. To date however, no studies have been published on the neural effects of LDX in moderate to severe BED.

It is widely recognised that cognition and behaviour arise from the dynamic interactions of distributed brain areas operating in networks. These large-scale networks can be imaged during functional tasks that evoke their activity, or during rest, when they tend to produce highly synchronised, very low frequency neuronal oscillations [25]. Analysis of resting-state functional magnetic imaging (rs-fMRI) can reveal much about the innate organisation of the brain, by demonstrating how brain regions, or nodes, are organised within networks, and how various networks are intrinsically connected with one another [26]. Applying a connectomic approach increases potential for providing new insights into the neural mechanisms of pharmacotherapy by providing comprehensive descriptions of these intrinsic connectivity patterns [27, 28].

There are currently no studies published on intrinsic connectivity patterns in BED relative to controls. As such, it is difficult to hypothesise precisely how LDX will effect neural connectivity in this group. Given the known dysfunction in the fronto-striatal regions relating to reward and inhibition in BED, it is assumed however that effective treatment with LDX will “normalise” activity and connectivity within these networks. We will also be able to examine relationships between treatment-driven neural changes and a large range of symptom measures and objective laboratory-based measures of cognition. These secondary meaures may also help to determine whether LDX exerts beneficial effects more broadly to non-food inhibitory control and other general cognitive improvements.

The primary aim of this study is to provide a comprehensive understanding of the neural mechanisms by which LDX improves symptoms of BED. We hypothesise that LDX will act by normalising connectivity within and between brain circuits responsible for reward and impulse control. Further, we hypothesise that a reduction in binge eating behaviours will correlate with normalised activity and connectivity of brain regions within reward and impulse control networks. Secondary aims include examining treatment-related changes in cognitive performance and other self-reported measures of symptoms and behaviour.

## Method

### Participants

Approximately 40 participants with BED and 22 healthy matched controls will be recruited into the study. Both BED and control participants must be between 18 to 40 years of age (inclusive), and have a BMI between 20 to 45 kg/m^2^ (inclusive). BED participants are required to have a diagnosis of moderate to severe BED. This is based on Module I of the *Structural Clinical Interview for DSM-5 Research Version* (SCID-5-RV) [29]. Consistent with LDX efficacy studies [18, 19], moderate to severe BED severity requires a minimum of three days of binge eating per week in the past month and a minimum score of 4 on the *Clinical Global Impression – Severity* scale (CGI-S) [30], a clinician-determined summary measure of a patient’s global functioning. The *MINI International Neuropsychiatric Interview Version 7.0.2 for DSM-5* [31] (MINI) is also used to assess comorbid psychiatric disorders to determine eligibility and better characterise the cohort. The full inclusion and exclusion criteria for BED and control participants are listed in Tables 1 and 2.

**Table 1:** Inclusion and exclusion criteria for BED participants

Criteria	List
Inclusion	• Age 18–40 years.
	• BED diagnosis, confirmed by the eating disorders module of the Structured Clinical Interview for DSM-5.
	• Moderate to severe BED, defined as the presence of binge eating frequency of ≥3 days/week in the month prior to the baseline assessment and a score of ≥4 on the clinical global impressions severity scale.
	• BMI of 20 – 45 kg/m^2^.
	• A study doctor has verified that it is medically and psychiatrically safe for their patient to commence LDX.
	• Fluent in English.
	• Have provided written informed consent.
Exclusion	• History of psychosis or mania.
	• Pregnant or breast-feeding women.
	• Current therapy with antipsychotics or noradrenaline reuptake inhibitors.
	• Current therapeutic intervention specific to treating eating behaviours and/or cognitions.
	• Cardiovascular disease, hypertension, use of monoamine oxidase inhibitors, or any other contraindications for psychostimulants.
	• History of substance abuse/dependence (excluding nicotine).
	• Previous suicide attempts or current suicidal ideation.
	• Known medical condition, disease or neurological disorder which might, in the opinion of investigator/s, interfere with the assessments to be made in the study or put BED patients at increased risk when exposed to optimal doses of the drug treatment.
	• Use of a psychostimulant in the 6 months prior to the study.
	• Inability to tolerate the MRI scanner due to physical or psychological factors.
	• History of physical brain injury or blow to the head that resulted in loss of consciousness for at least 10 min.

**Table 2:** Inclusion and exclusion criteria for control participants

Criteria	List
Inclusion	• Age 18–40 years.
	• BMI of 20 – 45 kg/m^2^.
	• Fluent in English.
	• Have provided written informed consent.
	• Current or previous diagnosis of an eating disorder or any other psychiatric diagnosis, including substance dependence.
Exclusion	
	• Pregnant or breast-feeding women.
	• Inability to tolerate the MRI scanner due to physical or psychological factors.
	• Known medical condition, disease or neurological disorder which might, in the opinion of investigator/s, interfere with the assessments to be made in the study or put subjects at increased risk.
	• History of physical brain injury or blow to the head that resulted in loss of consciousness for at least 10 min.
	• Prior treatment with any stimulant medication.

Participants are being recruited via referral by participating clinicians or self-referral due to online advertisements through Facebook, volunteer job sites, and webpages of universities in the Sydney metropolitan area.

### Overall study design

**Fig. 1:**
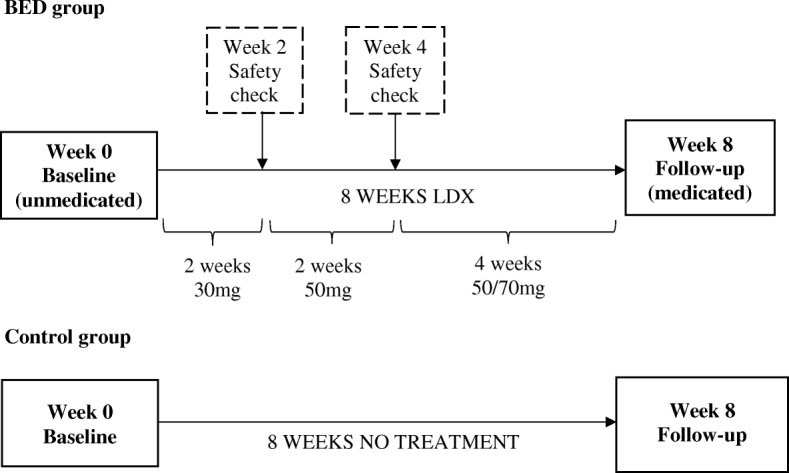
Study design and timeline of events for participants with Binge Eating Disorder (BED) and healthy controls

This study is a repeated-measures pre-post treatment design. Repeated-measures data from a control group is being collected at week 0 and week 8 for normative comparisons. Figure 1 outlines the timeline of events.

#### Screening

Potentially eligible participants complete an online screening questionnaire, followed by a more detailed telephone screen with a researcher. BED participants who appear eligible following the telephone screen undergo a medical history and safety assessment with a study clinician. The relevant prescription authority approvals are sought for each potential BED participant.

#### Pre-treatment baseline session (week 0)

During a 6 h testing session, all participants complete 1. a clinical interview and health check, 2. functional, structural and resting-state magnetic resonance imaging (MRI), 3. several self-report questionnaires relating to behavioural, attitudinal, cognitive and affective aspects of eating-disorder psychopathology, 4. a series of cognition-based tasks, and 5. a saliva sample for genetic analysis (optional). BED participants are provided with self-monitoring sheets and a script for their first course (4 weeks) of medication.

#### Clinical safety checks (week 2 and week 4)

After 2 weeks of treatment, BED participants have their blood pressure and heart rate measured by a study researcher or their General Practitioner. Participants are instructed to commence the increased dose of 50 mg/day if it is determined to be safe by the study clinician. After 4 weeks of treatment, participants meet with the study clinician for a comprehensive safety and dose check, including vitals, weight, drug compliance, adverse events and frequency of binge eating episodes. The clinician assesses the participants’ responsiveness to medication and symptom severity and determines whether the dose should remain at 50 mg/day or increase to 70 mg/day. Participants are then provided with a script for their remaining course (4 weeks) of medication. Participants are instructed to report any adverse effects or side effects as soon as possible, and may be advised to reduce the dose or stop the medication at any time.

#### Post-treatment session (week 8)

BED participants return the self-monitoring sheets provided during the baseline session and any unused tablets at the follow-up session. The majority of the assessments undertaken at session 1 are repeated at session 2 for both BED and control participants. The assessments/tasks that are not performed in session 2 are the SCID-5-RV and MINI, structural MRI and diffusion weighted imaging (DWI), and the Wechsler Test of Adult Reading [32] (WTAR). This session takes approximately 5 h to complete.

### Intervention

BED participants complete an 8 week course of LDX. The dose schedule for LDX is based on existing clinical practice and efficacy literature [18, 19]. All participants commence with 30 mg/day of LDX, and are advised to take it at the same time each morning. Participants are provided with self-monitoring sheets to help track their medication compliance.

After 2 weeks, participants will increase their dose to 50 mg/day if there are no abnormal changes to their blood pressure and heart rate. After 4 weeks, participants may continue with 50 mg/day or increase to 70 mg/day depending on their responsiveness to medication and symptom severity. Participants will remain on this dosage for another 4 weeks. At the week 8 follow-up session, participants will be required to return the completed self-monitoring sheets and any unused tablets. Participants with a compliance rate of less than 60% following a count of unused tablets will be viewed as non-compliant, and their data will be excluded from analysis.

### Neuroimaging acquisition

MRI scans are collected on a 3.0 Tesla Siemens Prisma magnet system (Siemens Healthineers, Erlangen, Germany) utilising a 64-channel phased-array head/neck coil and VE11C software. Specialised equipment from Thulborn Associates (Chicago, IL) and Lumina (Cambridge Research Systems, Kent, UK) is used to facilitate the fMRI data collection.

#### Resting-state fMRI

A resting-state echo-planar imaging (EPI) gradient echo (GRE) sequence is acquired with the following parameters: repetition time (TR) =1500 ms, echo time (TE) =33 ms, field of view (FOV) =255 mm, matrix = 104 × 104, flip angle = 85°, acceleration factor (IPAT) = 6, phase-encoding direction = anterior to posterior. Sixty contiguous axial/oblique slices with a slice thickness of 2.5 mm are acquired to cover the whole brain. Eight dummy scans are acquired at the start of every acquisition to allow magnetization to stabilize to steady state. A total of 320 measurements are subsequently acquired during the Resting State period, resulting in a total scan time of 8 minutes and 12 s.

#### Structural T1 MPRage

A high-resolution isotropic 3-dimensional (3D) T1-weighted volume dataset is subsequently acquired in the sagittal plane using an inversion recovery (IR)-prepared spoiled gradient echo (MP-RAGE) sequence with the following parameters; TR = 2400 ms; TE = 2.21 ms; time following inversion pulse (TI) = 900 ms; FOV = 256 mm; matrix = 288 × 288; flip angle = 8°; multi-slide mode = single shot; IPAT = 2; phase-encoding direction = A/P. A total of 192 slice locations are acquired with an effective slice thickness of 0.9 mm covering the whole brain in a scan time of 6 minutes and 23 s. This 3D dataset is collected for use in a unified segmentation approach for normalization of the fMRI data to standard space.

#### Task-based fMRI

PsychoPy software (version 1.85.3) is used for the delivery of functional stimulus. Two functional tasks are performed during image acquisition utilising a GRE EPI sequence with the following parameters: TR = 3000 ms, TE = 20 ms, FOV = 240 mm, matrix = 104 × 104, flip angle = 90°, IPAT = 2, phase-encoding direction = A/P. During each task, 55 contiguous axial/oblique slices with a slice thickness of 2.5 mm are acquired to cover the whole brain. For the Pavlovian-Instrumental Transfer (PIT) task, 284 measurements are acquired during the task period, resulting in a total scan time of 14 minutes and 27 s. For the Devaluation task, 120 measurements are acquired during the task period, resulting in a total scan time of 6 minutes and 15 s.

#### Diffusion weighted imaging

A multi-shell EPI DTI technique with 3 separate acquisitions of 64 axial slices each is also utilized with the following parameters: TR =3500 ms: TE = 71 ms: matrix = 110 × 110: FOV = 260 mm: slice thickness = 2.4 mm; IPAT = 2: phase-encoding direction = A/P. Acquisition 1 (4 mins 23 s) is 69 directions with b-values of 0 and 2800 s/mm^2^; acquisition 2 (3 mins 16 s) is 50 directions with b-values of 0 and 2000 s/mm^2^; acquisition 3 (2 mins 6 s) is 30 directions with b-values of 0 and 1000 s/mm^2^. A pair of gradient reversal sequences (AP and PA k-space trajectories) are also acquired for distortion correction.

## Measures

### Clinical measures

#### Number of binge eating days per week

The number of binge eating days will be recorded pre- and post- treatment as a measure of BED symptom improvement.

#### Clinical global impressions – Severity [30]

The CGI-S score will also be recorded pre- and post- treatment as a measure of BED symptom improvement.

### Neuroimaging measures

#### Resting-state functional magnetic resonance imaging (rs-fMRI)

During the resting-state scan, participants are asked to “keep your eyes open and try to keep them on the cross. Try to remain still but not fall asleep”. A key measure extracted from this data will be connectivity strength, which is derived from the degree of synchronisation of very low frequency neuronal oscillations between each node across time. Descriptive features of the neuronal networks derived from graph-theory will also be produced, such as small-worldness, modularity and network efficiency [25].

#### Task-based functional magnetic resonance imaging (fMRI) – Pavlovian-instrumental transfer and devaluation Tests [33]

Outside of the scanner, participants learn to use specific actions to liberate snack foods from a vending machine. They also learn about the reward value of the foods, changes in reward value, and the relationship between various predictive stimuli and food delivery. Within the scanner, the PIT Test aims to evaluate the ability of subjects to use experienced or predicted value to guide goal-directed actions. Subsequently, the reward value of the food is manipulated via disgust (a video of cockroaches on the food). The Devaluation test is used to assess the ability of subjects to adjust their food-seeking actions based on changes in current reward value.

#### Diffusion weighted imaging

This scan of white matter structure allows for analysis of the structural architecture of the brain and the integrity of white matter pathways. Measures of white matter integrity, produced using tract-based spatial statistics, will include fractional anisotropy (FA) and mean diffusivity (MD). Probabilistic tractography will be conducted to examine the white matter connectome and assess connectivity strength between specific nodes.

### Symptom-related measures

Self-report and interview-based questionnaires detailed below will be administered at both baseline and week 8 for BED participants, and at baseline only for control participants.

### Eating-related behaviours and cognition

#### Self-report measures

The Three-Factor Eating Questionnaire [34] assesses 3 cognitive and behavioural aspects of eating: dietary restraint, disinhibition and hunger. The Binge Eating Scale [35] measures the behavioural, emotional and cognitive symptoms associated with binge eating. The Eating Disorders Examination Questionnaire [36] measures eating disorder psychopathology in the past month related to restraint, eating concern, shape concern, and weight concern, as well as behavioural symptoms related to these concerns. The Loss of Control Over Eating Scale [37] is a measure of multiple aspects of loss of control whilst eating.

#### Researcher-administered measures

The Yale-Brown Obsessive Compulsive Scale Modified for Binge-Eating is a modified form of the Yale-Brown Obsessive Compulsive Scale [38] that has been used in prior randomised controlled trials of LDX in BED [17–19] and measures the obsessiveness of binge eating thoughts and compulsiveness of binge eating behaviours.

### Impulsivity

The Barratt Impulsiveness Scale [39] is a self-reported questionnaire which measures personality and behavioural aspects of impulsivity in individuals.

### Other psychological symptoms

The Adult attention-deficit hyperactivity disorder (ADHD) Self-Report Scale [40] assesses the frequency of recent symptoms of ADHD in adults. The researcher-administered Hamilton Rating Scale for Depression [32] and Hamilton Anxiety Rating Scale [41] assess the severity of depression and anxiety symptoms, respectively.

### Quality of life

The self-reported WHO Quality of Life-BREF is a shortened form of the WHO Quality of Life-100 assessment [42] and is commonly used in epidemiological studies and clinical trials. It measures four broad domains related to quality of life: physical health, psychological health, social relationships, and the environment.

### Laboratory-based cognition

With the exception of the WTAR (baseline only), the tasks detailed below will be administered at both baseline and week 8 for all participants.

1. *Wechsler Test of Adult Reading* (WTAR) [43]. A short word reading task that provides a measure of premorbid intelligence.
2. *GoNoGo* [44]. In this task, the colour of the word ‘PRESS’ is frequently presented in green (Go) and infrequently in red (No Go). The participant is required to inhibit circle-tapping responses on red. This task measures target detection rate, response time, errors of commission and omission. The GoNoGo is a test of behavioural inhibition.
3. *Stroop test* [45]. There are two sections to this test. In the first, the participant is required to indicate the colour that the written word spells (and not the incongruent ink colour that the word is written in). In the second section, the subject is asked to name the ‘ink’ colour a word is written in (and not read the actual word). The ‘interference’ experienced from the written word is called the ‘Stroop’ effect. The interference arises from the fact that reading is a highly over-learned skill and occurs automatically unless there is a sustained attentional focus to suppress the reading response. This assesses selective attention capacity and cognitive processing speed.
4. *Monetary Incentive Delay (MID) Task* [46]. The MID task requires an individual to react to a target stimulus presented after an incentive cue to win or to avoid losing the indicated reward. In doing so, this paradigm allows the detailed examination of different stages of reward processing like reward prediction, anticipation, outcome processing, and consumption.
5. *Value-modulated attentional capture* [47]. In this visual search task, certain stimuli signal the magnitude of available reward, but reward delivery is not contingent on responding to those stimuli. Indeed, any attentional capture by these critical distractor stimuli leads to a reduction in the reward obtained. Attentional capture is measured using eye-tracker equipment. Any counterproductive capture by task-irrelevant stimuli is important because it demonstrates how external reward structures can produce patterns of behavior that conflict with task demands, and similar processes may underlie problematic behavior directed toward real-world rewards. This measures reward sensitivity in involuntary attentional capture.

### Genetic analysis

BED participants will be given the option to provide a sample of their saliva for genetic analysis. Separate informed consent is obtained for participants who opt-in. The sample will be used to extract DNA and investigate single-nucleotide polymorphisms (e.g. DRD4, DRD5) relating to the pharmacogenetics of LDX.

### Primary outcomes

Resting-state intrinsic connectivity metrics and the number of binge eating episodes, pre- and post- LDX treatment, are the primary outcome measures of this study.

### Secondary outcomes

LDX treatment effects on activation and connectivity evoked during the PIT and Devaluation Tests, and behavioural performance on these tasks, are key secondary outcomes. Analyses will also be undertaken to examine LDX treatment effects on all symptom-related measures, and cognitive tasks outlined above. Potential side-effects (e.g. change in BMI) will be examined as covariates in analyses of treatment effects.

### Supplementary outcomes

Comparisons will be made between the pre-treatment BED group and healthy controls on the full range of measures to better characterise symptoms, cognition and neural function in BED.

### Sample size calculation

Forty individuals with BED will be recruited for this study. At the Brain Dynamics Centre, we have a record of high treatment trial retention rates (e.g. Methylphenidate trial in child and adolescent ADHD, 98%) and minimal neuroimaging data being discarded due to excessive motion (e.g. in young adults with depression, 2–3 individuals/ 40 recruited). Based on this history, we assume there will be a final sample size of approximately 35 for the primary outcome measures. This far exceeds previous imaging studies in BED (range of 11–22 participants per study) [7, 12, 13]. In addition, the pre- post treatment design enhances power due to the use of *within subjects* rather than *between groups* comparisons. A sample size of 35 should provide approximately 80% power to observe a medium effect size of 0.5 for a two-sided statistical analysis at an alpha value of 0.05. Studies examining fMRI connectivity changes between methylphenidate (a related stimulant) and placebo have observed strong effects with sample sizes much lower than that proposed here (e.g. *n* = 12) [48–50].

### Data analysis

Only the data from individuals with a medication compliance rate of ≥60% will be included in primary outcome analyses. For all analyses, a *p* value of < 0.05 will be considered statistically significant and relevant corrections for multiple comparisons will be applied.

Framewise displacement—movement of the head from one volume/timepoint to the next—and scaled signal intensity differences between contiguous volumes, will be calculated for each rs-fMRI time series during the quality control process. Temporal masks will be applied to censor volumes including motion, and participant data will be excluded from analyses if more than 1/3 of the time course data requires temporal masking.

Functional connectomics involves the study of brain connectivity, first by identifying the nodes (distinct brain regions) and subsequently estimating the functional connections (network edges) between these nodes [51]. Both voxel-wise and brain atlas parcellation approaches (e.g. Gordon parcellations) [52] will be utilised to designate nodes. Data-driven, whole-brain analyses using methods such as multivariate distance matrix regression [53] and network based statistics [54] will then be applied to rs-fMRI data to characterise pharmacological effects across the functional connectome, and assess correlations with symptom measure changes. Using a similar approach to Yang et al. [55], the results of data-driven analyses will be followed up using seed-based functional connectivity analyses. Connectomic measures will be computed for BED participants at baseline and post-treatment. A paired-sample t-test will be performed to compare baseline and post-treatment connectivity profiles to confirm the treatment effect. To examine any normalisation effects of LDX in BED participants, two independent sample t-tests will be conducted to compare functional connectivity between healthy controls, and the BED group at baseline and post-treatment, respectively. To examine the association of changes in functional connectivity and clinical improvement, we will correlate change scores (Δ = BED post-treatment – BED baseline) of functional connectivity with changes in the number of binge-eating episodes.

A similar approach to that described above will be used to assess treatment-related changes in secondary outcome measures, and to examine relationships between these measures. Two group repeated-measures analyses will also be conducted to compared change scores (Δ = week 8 – week 0) between BED and control groups across tasks, to account for practise effects. For supplementary outcomes, two-sample t-tests will be performed between the pre-treatment BED and control groups.

Statistical parametric mapping (SPM12) (www.fil.ion.ucl.ac.uk/spm) and FSL (http://www.fmrib.ox.ac.uk/fsl) will be used for preprocessing neuroimaging data. Networks statistics will be computed using R package connectir (http://czarrar.github.io/connectir) and Network-Based Statistic (NBS) (https://www.nitrc.org/projects/nbs/). General statistics will be conducted using R (www.r-project.org/), SPSS (https://www.ibm.com/au-en/products/spss-statistics), and SPM.

## Discussion

The combination of neuroimaging and laboratory-based cognitive measures will provide a sophisticated and comprehensive explanation of the neural mechanisms by which LDX improves BED symptomology. The repeated measures design will enable participants to act as their own controls in assessing whether LDX changes interconnectivity and integration, particularly in brain regions responsible for reward sensitivity and impulse control. We can also assess whether interconnectivity and integration post-treatment more closely resembles healthy controls, relative to pre-treatment.

A limitation of this study is that we are unable to draw absolute conclusions on the causality of LDX in our results as the design does not include a placebo arm. There are confounding factors that could contribute to changes in neural connectivity and binge eating symptomology, for instance, use of a self-monitoring diary, clinician-patient interaction during study assessments and other social and environmental factors. While we may not be able to attribute changes in BED symptomology and neural connectivity solely to the effects of LDX, the results are more generalisable to a real-world setting where these factors exist and contribute to treatment effects. As this study is not designed to assess treatment efficacy, accounting for placebo effect is not required. Another limitation is that the primary measure of binge-eating behaviour, the number of binge eating events, is based on self-report, which relies on memory and unbiased reporting for accuracy. Finally, more information is required on the longer term efficacy and safety implications of LDX treatment, however this is beyond the scope of the current study and should be a direction for future research.

## Conclusion

Despite LDX being the only approved pharmaceutical method of treatment for moderate to severe BED in Australia, the exact neurobiological mechanisms of its action are unknown. BED is often under-diagnosed and under-treated, and those who do seek treatment can be non-responsive to first-line psychological forms of treatment. This study will utilise novel neuroimaging techniques that will examine changes in brain interconnectivity and integration with far greater detail and accuracy than ever before. This will contribute to determining the neurobiological basis for BED, and how LDX promotes changes within those neural mechanisms to reduce BED symptomology.

## Data Availability

The datasets used and/or analysed during the current study are available from the corresponding author on reasonable request.

## Abbreviations

3D: 3-dimensional
A/P: anterior to posterior
ADHD: Attention-deficit hyperactivity disorder
BED: Binge eating disorder
BMI: body-mass index
CGI-S: Clinical global impression – severity
DA: Dopamine
EPI: Echo-planar imaging
FA: Fractional anisotropy
FDA: Food and drug administration
fMRI: Functional magnetic resonance imaging
GRE: Gradient echo
HREC: Human Research Ethics Committee
ICH: International conference on harmonisation of technical requirements for pharmaceuticals for human use
IPAT: Acceleration factor
IR: Inversion recovery
LDX: Lisdexamfetamine
MD: Mean diffusivity
MID: Monetary incentive delay
MINI: The MINI international neuropsychiatric interview version 7.0.2 for DSM-5
MP-RAGE: Magnetization prepared rapid acquisition with gradient echo
MRI: Magnetic resonance imaging
NA: Noradrenaline
NBS: Network-based statistic
NHMRC: National Health and Medical Research Council
PET: Positron emission tomography
PIT: Pavlovian-instrumental transfer
rs-fMRI: Resting-state functional magnetic imaging
SCID-5-RV: Structural clinical interview for DSM-5 research version
SPM12: Statistical parametric mapping
TE: Echo time
TR: Repetition time
